# Targets downstream of Cdk8 in *Dictyostelium *development

**DOI:** 10.1186/1471-213X-11-2

**Published:** 2011-01-21

**Authors:** David M Greene, Gareth Bloomfield, Jason Skelton, Alasdair Ivens, Catherine J Pears

**Affiliations:** 1 Biochemistry Department, Oxford University, South Parks Road, Oxford OX1 3QU UK; 2 MRC Laboratory of Molecular Biology, Hills Road, Cambridge, CB2 2QH, UK; 3 The Wellcome Trust Sanger Institute, Wellcome Trust Genome Campus, Hinxton, Cambridge, CB10 1SA, UK; 4 Fios Genomics Ltd, ETTC, King's Buildings, Edinburgh, EH9 3JL, UK

## Abstract

**Background:**

Cdk8 is a component of the mediator complex which facilitates transcription by RNA polymerase II and has been shown to play an important role in development of *Dictyostelium discoideum*. This eukaryote feeds as single cells but starvation triggers the formation of a multicellular organism in response to extracellular pulses of cAMP and the eventual generation of spores. Strains in which the gene encoding Cdk8 have been disrupted fail to form multicellular aggregates unless supplied with exogenous pulses of cAMP and later in development, *cdk8*^- ^cells show a defect in spore production.

**Results:**

Microarray analysis revealed that the *cdk8*^- ^strain previously described (*cdk8*^-^_HL_) contained genome duplications. Regeneration of the strain in a background lacking detectable gene duplication generated strains (*cdk8*^-^_2_) with identical defects in growth and early development, but a milder defect in spore generation, suggesting that the severity of this defect depends on the genetic background. The failure of *cdk8*^- ^cells to aggregate unless rescued by exogenous pulses of cAMP is consistent with a failure to express the catalytic subunit of protein kinase A. However, overexpression of the gene encoding this protein was not sufficient to rescue the defect, suggesting that this is not the only important target for Cdk8 at this stage of development. Proteomic analysis revealed two potential targets for Cdk8 regulation, one regulated post-transcriptionally (4-hydroxyphenylpyruvate dioxygenase (HPD)) and one transcriptionally (short chain dehydrogenase/reductase (SDR1)).

**Conclusions:**

This analysis has confirmed the importance of Cdk8 at multiple stages of *Dictyostelium *development, although the severity of the defect in spore production depends on the genetic background. Potential targets of Cdk8-mediated gene regulation have been identified in *Dictyostelium *which will allow the mechanism of Cdk8 action and its role in development to be determined.

## Background

The serine/threonine kinase Cdk8 is a regulator of transcription through its association with the mediator complex [1]. This complex was originally identified as an activity that was required to allow RNA polymerase II to perform regulated transcription *in vitro*. Purification has revealed it be a large multi-protein complex with varying composition. The presence of a submodule containing Cdk8 and its protein partner cyclin C has been proposed to be a mechanism to regulate mediator activity, responsible for both activation and inhibition of transcription through phosphorylation of either the C terminal domain of RNA polymerase II (CTD) or of gene-specific transcription factors. The yeast orthologues of Cdk8 (Srb10) and cyclin C (Srb11) were identified genetically as suppressors of defects caused by truncation of the CTD, consistent with a role in regulation of transcription. Microarray analysis suggests that yeast lacking Srb10 show altered expression of around 3% of genes [2]. Orthologues of Cdk8 are apparent in all eukaryotes, but the mechanisms of regulation are not well defined. In *S. cerevisiae*, proteolysis of the cyclin C orthologue has been proposed in response to stresses such as oxidative stress [3], but this has not been reported in other systems.

In mammalian cells Cdk8 activity has been implicated in regulation of growth through its overexpression in tumour cells [4], as well as in development. Cdk8 plays an important role in the Notch signalling pathway as recruitment of Cdk8 to the promoter of a developmentally regulated gene (HES1) causes hyperphosphorylation of the intracellular domain of the Notch transcription factor, promoting its degradation and leading to subsequent down regulation of transcription of HES1 [5]. Cdk8-dependent regulation of transcription factor stability has also been reported in *S. cerevisiae *where phosphorylation of Ste12 and GCN4 by Srb10 promotes their degradation [6,7]. Consistent with a role in development, Cdk8 shows tissue specific expression during zebra fish development and disruption of genes encoding components of the mediator sub-complex containing Cdk8 cause developmental defects in *C. elegans*, *Drosophila *and Arabidopsis [8-10].

Previous reports have implicated Cdk8 activity in development of *Dictyostelium *amoebae [11,12] which feed and proliferate as single cells but, upon starvation, undergo a developmental life cycle [13]. Starving cells start to secrete pulses of cAMP that acts as a chemoattractant for the surrounding cells which aggregate to form a multicellular organism. This aggregate undergoes a series of morphogenetic changes leading to the generation of a fruiting body in which around 80% of the cell differentiate into spore cells while the remaining cells differentiate into stalk cells to support the spore head above the substratum. *Dictyostelium *strains in which the gene encoding Cdk8 have been disrupted grow poorly in shaking suspension and fail to aggregate unless supplied with exogenous pulses of cAMP [11,12]. The cells also fail to express a number of genes associated with early development, including the gene encoding the adenylyl cyclase (*aca*) responsible for cAMP generation during aggregation, which could explain the failure to aggregate. We further reported that for the *cdk8*-null strain generated in our lab (henceforth called *cdk8*^-^_HL_) pulsing with exogenous cAMP could rescue the early aggregation defect. If these cells were then plated on a surface, they went on to form aberrant structures with a defect in the ability to generate mature spore cells. This defect could be rescued by expression of Cdk8 but not by a kinase-dead version, implicating the kinase activity in this failure of development [11]. However, although early aggregation defects were identical, *cdk8*^- ^cells generated in a different genetic background did give rise to viable spores [12].

Here we report that microarray analysis revealed genome duplications in the *cdk8*^-^_HL _cells, which may explain the phenotypic differences in different genetic backgrounds. Subsequent regeneration of the strain in a parent lacking detectable genomic duplications confirms the defects in aggregation and an important role for Cdk8 in spore cell differentiation, although milder than initially reported. Comparison of the protein expression profile of parental and the new strain of *cdk8*^- ^(*cdk8*^-^_2_*) *cells revealed potential targets for direct regulation by Cdk8 which will facilitate analysis of the targets of Cdk8 in *Dictyostelium *which lead to defects in growth and development.

## Results

### Microarray analysis of *cdk8*^-^_HL _cells

**Figure 1:**
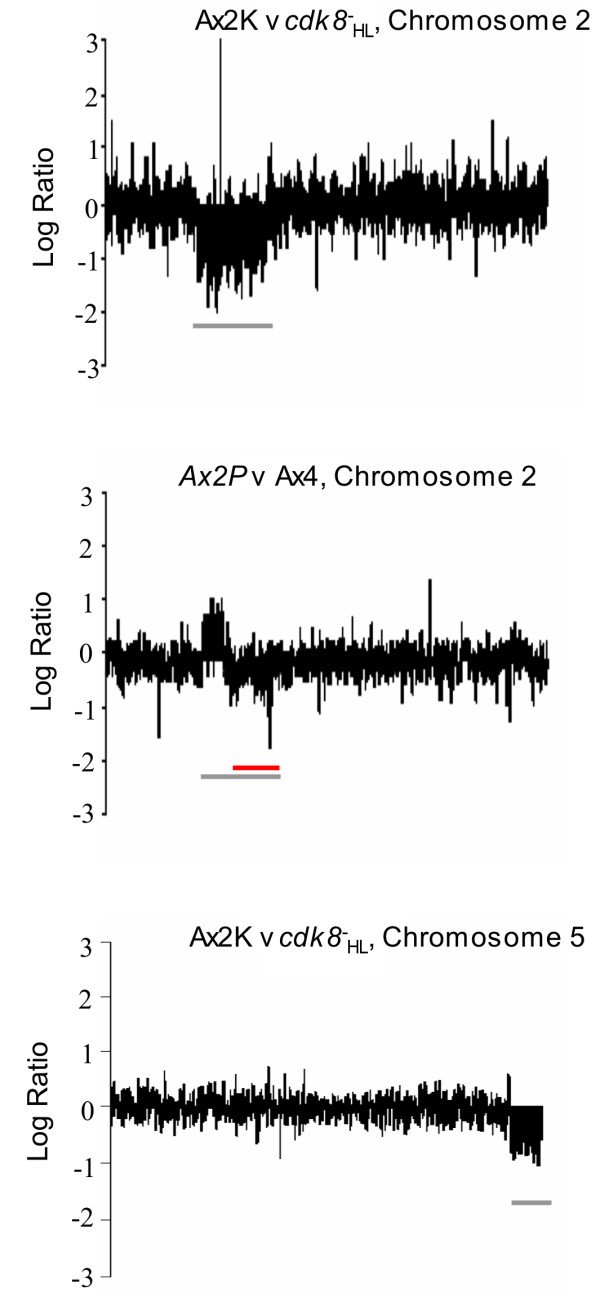
**Microarray comparisons of genomic DNA from Ax2, Ax4 and *cdk8*^-^_HL _strains**. The log(2)ratio of abundance of each gene in each comparison is plotted against the position of that gene on the chromosome. Ax2P is the parental strain used to generate *cdk8*^-^_HL _and Ax2K is the Ax2 strain with minimal genomic duplications [14]. Shown are comparison of genes on Chromosome 2 between Ax2K and *cdk8*^-^_HL_, and between Ax2P (the parent of *cdk8*^-^_HL_) and Ax4, and a comparison of genes on chromosome 5 for Ax2K and *cdk8*^-^_HL_. The approximate position of the putative *cdk8*^-^_HL _duplications relative to Ax2K are marked with grey horizontals bars. Logratios of approximately 1 or -1 indicate 2-fold changes in copy number (duplications); logratios around zero indicate equal copy number. The known Ax4 duplication is marked with a red horizontal bar; logratios in this region average approximately zero in this comparison show that both strains have the same copy number here. Ax2P has a longer duplication than that apparent in Ax4, though in the same region, as represented by the grey bar in the middle panel. The *cdk8 *gene is found on chromosome 1 and so its loss is not apparent on the comparisons shown here.

In order to investigate differences in the reported phenotypes of strains containing disruption of the *cdk8 *gene in *Dictyostelium *and in light of the recent realisation that many laboratory strains of *Dictyostelium *contain genomic duplications [14], we investigated whether the *cdk8*^- ^cells previously isolated in our laboratory, *cdk8*^-^_HL_, and their parent, Ax2P, contained genomic duplications. This analysis was carried out by microarray to compare the relative copy number of genes in genomic DNA isolated from these strains and from the strain Ax2K which is free of detectable duplications [14]. Comparison of both *cdk8*^-^_HL _and its parental Ax2 line (Ax2P) with Ax2K [14] revealed a large duplication on chromosome 2 in both *cdk8*^-^_HL _and Ax2P (Figure 1 and data not shown). This analysis also revealed a further duplication on chromosome 5 within *cdk8*^-^_HL _cells, which was not present in the parent Ax2P. A duplication on chromosome 2 in the region duplicated in Ax2P and its derivative, has previously been reported for another strain of *Dictyostelium*, Ax4 [15]. Comparison of the parental Ax2P gDNA with gDNA extracted from strain Ax4 showed that the novel chromosome 2 feature in the former strain spans the previously described 750 kb duplication in Ax4, and extends approximately a further 400 kb on one side of it. In view of the chromosomal duplications identified in the original *cdk8*^-^_HL _strain, independent null strains were generated in an Ax2 background, using the Ax2K cells with no detectable duplications [14]. Three independent strains were identified in which the *cdk8 *gene had been disrupted and all showed identical phenotypes to each other. In order to determine whether the phenotypes described for the original *cdk8*^-^_HL _strain were dependent on the genome duplications we carried out a phenotypic analysis of the newly derived *cdk8*^-^_2 _cells. They showed identical defects in growth in shaking suspension to the original strain (data not shown). They also failed to form aggregates on starvation, but this defect could be rescued by exogenous pulses of cAMP, as reported for the original strain, and showed similar defects in transcription of early developmental genes such as a failure to switch off expression of *cprD *and a failure to induce expression of early developmental genes such as *pkaC*, *aca *and *carA *on starvation (data not shown) [11,12]. These results suggested that the early developmental phenotype was not influenced by the genome alterations in the original *cdk8*^-^_HL _strain.

### Late developmental phenotype of the *cdk8*^-^_2 _strain

**Figure 2:**
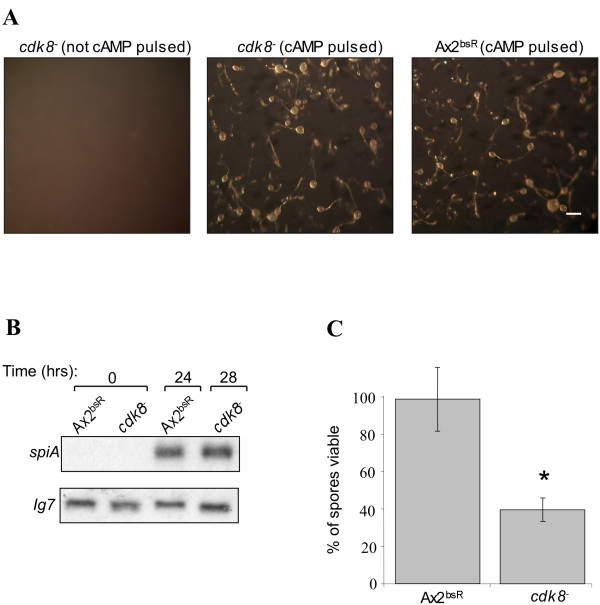
**Late developmental phenotype of *cdk8*^-^_2 _cells**. **(A) **Cells were developed in KK_2 _buffer at 1 x 10^7^cells/ml with or without cAMP pulsing (50 nm cAMP added to the suspension every 5 minutes). Each strain was shaken for 6hrs at 150 rpm at 22°C before being spread onto filters at a density of 3 x 10^6 ^cell/cm^2^. Photographs were taken after 28hrs. The scale bar in the right hand panel represents 200 μm. **(B) **Expression of *SpiA *in *cdk8*^-^_2 _cells. Fruiting bodies formed after cAMP pulsing were harvested after 24hrs or 28 hrs. RNA was extracted from these samples and resolved on a 1% formaldehyde gel, transferred to a nylon membrane and probed with a ^32^P labelled fragment of the *spiA *gene. The blot was reprobed with a ^32^P labelled fragment of the *IG7 *gene so as to control for loading. **(C) **Viability of *cdk8*^-^_2 _spores. Fruiting bodies were formed by developing cells in shaken suspension with cAMP pulsing prior to plating. Equal numbers of spores from each strain were treated with heat and detergent and spread onto a bacterial lawn. Colonies resulting from hatching of viable spores were counted after 4-5 days. Each bar represents the mean (±standard deviation) of three independent experiments. Results showing statistically significant difference from Ax2^bsR ^are marked with a * (p < 0.05 by student t-test).

In order to examine the newly generated *cdk8*^-^_2 _strain for the presence or absence of the late developmental phenotype, cells were suspended in KK_2 _buffer and pulsed with 50 nM cAMP every 5mins for 6hrs, before being harvested, washed and plated on KK_2 _agar. Unlike the *cdk8*^-^_HL _strain, the *cdk8*^-^_*2 *_cells formed phenotypically normal fruiting bodies, although culmination occurred 3-4hrs later than in the Ax2^bsR ^control strain (Figure 2A). Ax2^bsR ^was created by random insertion of the vector designed for disruption of the *cdk8 *gene into the genome of Ax2K. Upon culmination, the *spiA *gene (a gene induced late in spore differentiation and found to be expressed at reduced levels in the original *cdk8*^-^_HL _cells) was expressed at similar levels in both the *cdk8*^-^_2 _and Ax2^bsR ^strains (Figure 2B).

### Viability of *cdk8*^-^_2 _spores

In order to investigate whether the spores generated by the *cdk8*^-^_2 _strain were viable, their ability to germinate after heat and detergent treatment was tested. Fruiting bodies formed by the mutant and control strains were harvested and disaggregated. An equal number of spores from each strain were exposed to detergent and heat treatment in order to destroy any non-viable spores and undifferentiated amoebae. The number of surviving spores from each strain that were capable of germination was determined by spreading dilutions onto bacterial lawns and counting the resultant colonies.

This analysis revealed that *cdk8*^-^_2 _spores were less viable than those of the Ax2^bsR ^strain. Whilst 98% of spores from the control strain were able to germinate after heat and detergent treatment, this was true for only 40% of *cdk8*^-^_2 _spores (Figure 2C). This defect in spore formation phenotype is much milder than that of the previous *cdk8*^-^_HL _strain in which virtually no resistant spore cells were formed [11]. This data suggests that, although the phenotype was exaggerated in the duplication-containing background, there is a consistent defect in spore cell formation in the absence of *cdk8*.

### Requirement for kinase activity of Cdk8

**Figure 3:**
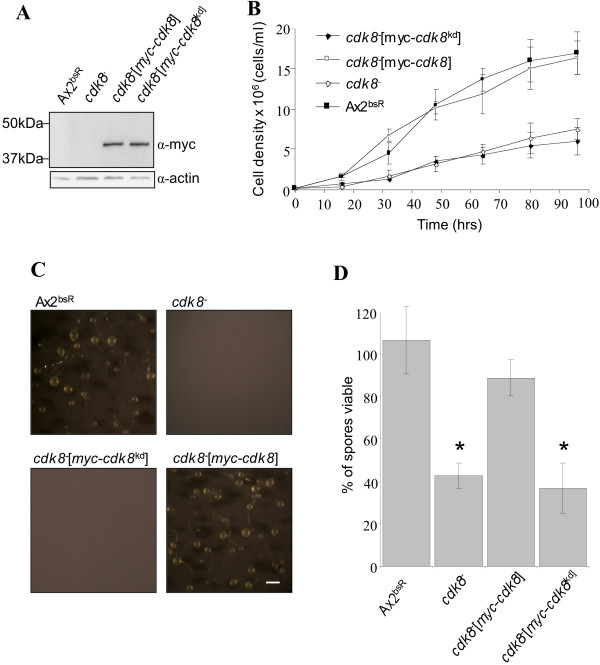
**Complementation of *cdk8***^-^_**2 **_**growth and aggregation defects**. **(A)** Expression of the myc-Cdk8 and myc-Cdk8^kd ^proteins was confirmed by western blotting. Samples were collected from vegetatively growing cells of each strain and resolved on a 12% SDS-PAGE gel, transferred to a nitrocellulose membrane and probed with the antibodies indicated. The blot was reprobed with an α-actin antibody to control for loading. **(B) **Complementation of slow-growth phenotype. Growth in HL-5 medium was assessed by counting cell number. The *cdk8*^-^_2 _and *cdk8*^-^_2_[*myc-cdk8*kd] strain eventually grew to a density of 2 x 10^7 ^cells/ml after 7-8days (data not shown). **(C) **Complementation of aggregation phenotype. Cells were developed on filters at 3 x 10^6 ^cells/ml at 22°C. Photographs were taken after 4 days. The scale bar in the bottom right hand panel represents 200 μm. **(D) **Complementation of spore viability defect. Fruiting bodies formed after developing with cAMP pulsing were harvested and spores assessed for viability as in Figure 2. Results showing statistically significant difference from Ax2^bsR ^are marked with a * (p < 0.05 by student t-test).

The *cdk8*^-^_2 _strain was transformed with an extrachromosomal vector to drive expression of an epitope tagged version of Cdk8 (myc-Cdk8) from a semi-constitutive actin15 promoter (pDXA[*act15*::myc-*cdk8*) and a version containing a point mutation (pDXA[*act15*::myc-*cdk8*^*kd*^]) to create a kinase deficient form of the same protein (myc-Cdk8^kd^) [11]. The expression of each Cdk8 protein was confirmed by western blot (Figure 3A). Expression of the myc-Cdk8 protein in *cdk8*^-^_2 _cells resulted in rescue of all the observed growth and developmental phenotypes. In contrast, the *cdk8*^-^_2_[myc-*cdk8*^*kd*^] strain grew at a rate comparable to the *cdk8*^-^_2 _strain and did not form aggregates upon starvation (Figures 3B and 3C). The *cdk8*^-^_2 _[myc-*cdk8*] spores exhibited a similar viability (89%) to those of the Ax2^bsR ^strain (104%) implying that expression of the myc-Cdk8 protein complemented the late developmental phenotype (Figure 3D). In contrast, the *cdk8*^-^_2_[myc-*cdk8*^*kd*^] spores exhibited a similar viability (43%) to those of the *cdk8*^-^_2 _strain (39%). These observations implied that the phenotypes observed in the *cdk8*^-^_2 _strain were directly attributable to a loss of Cdk8 kinase activity.

### Overexpression of *pkaC *in *cdk8*^-^_2 _cells

**Figure 4:**
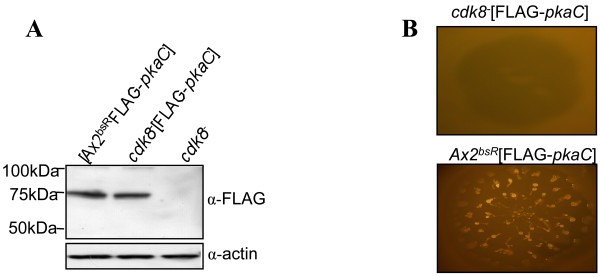
**Expression of constitutively active PKA in *cdk8*^-^_2 _cells**. **(A) **Western analysis was used to confirm expression of the FLAG-PKAC protein in the Ax2^bsR^[FLAG-*pkaC*] and *cdk8*^-^_2_[FLAG-*pkaC*] cell lines. Samples were collected from vegetatively growing cells of each strain and resolved on a 12% SDS-PAGE gel, transferred to a nitrocellulose membrane and probed with the α-FLAG antibody. The blot was reprobed with an α-actin antibody to control for loading. **(B) **Developmental phenotype of the Ax2^bsR^[FLAG-*pkaC*] and *cdk8*^-^_2_[FLAG-*pkaC*] strain. Cells were spotted onto a lawn of *K. aerogenes *and incubated at 22°C. Photographs were taken after 5 days. Each colony is approximately 1 cm in diameter.

The early developmental phenotype of the *cdk8*^-^_2 _cells suggested a potential gene target for regulation by Cdk8 activity. The cAMP dependent protein kinase (PKA) enzyme is vital for both aggregation and formation of spore cells (reviewed in [16]). Many aggregation-deficient strains which can be rescued by exogenous pulses of cAMP can also be rescued by restoring expression of the catalytic subunit of PKA, *pkaC*. As no expression of *pkaC *could be detected in *cdk8*^- ^cells ([11] and data not shown), it was hypothesised that this may be responsible for the defect in aggregation. In order to address this question, the pDXA[*act15::*FLAG-*pkaC*] plasmid which expresses the PKA-C protein with an N-terminal FLAG-tag was transformed into the *cdk8*^-^_2 _and Ax2^bsR ^to create the *cdk8*^-^_2_[FLAG-*pkaC*] and Ax2^bsR^[FLAG-*pkaC*] strains. Cell extracts from these strains were analysed by western blot to confirm expression of the 75kDa FLAG-PKA-C protein in both strains (Figure 4A).

Both strains were developed alongside the parental Ax2^bsR ^and *cdk8*^-^_2 _strains. It has previously been shown that over expression of PKA-C results in more rapid completion of the developmental cycle [17]. As the Ax2^bsR^[FLAG-*pkaC*] strain was observed to achieve culmination more rapidly than the parental Ax2^bsR ^strain (data not shown) it was concluded that a functional PKA-C protein was being expressed. However, expression of this protein in the *cdk8*^-^_2_[FLAG-*pkaC*] strain did not result in a rescue of the *cdk8*^-^_2 _aggregation defect (Figure 4B) suggesting that low levels of *pkaC *expression were not solely responsible for this phenotype.

### Identification of proteins with altered expression levels in *cdk8*^- ^cells

**Figure 5:**
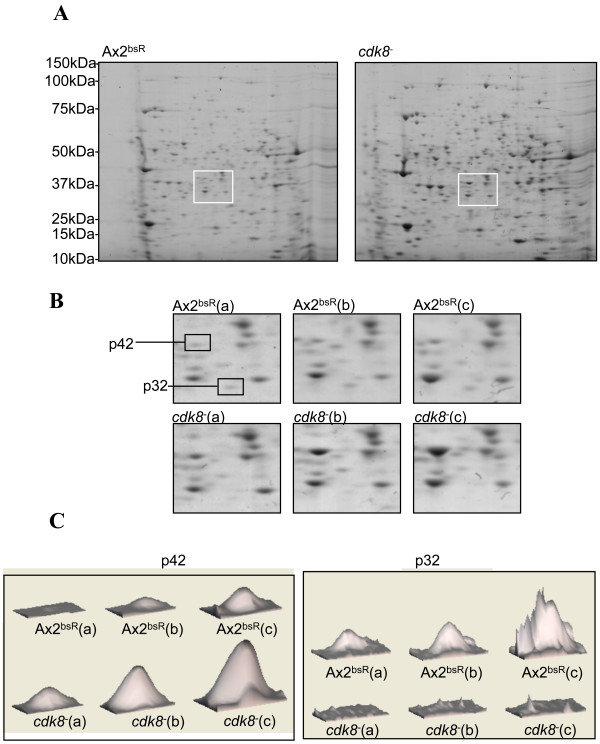
**Proteomic Comparison of Ax2^bsR ^and *cdk8*^-^_2 _cells**. **(A) **Aliquots containing 100 μg of soluble protein from whole-cell extracts of growing cells were acetone precipitated and resuspended in 125 μl of sample buffer. The samples were loaded onto nonlinear immobilized pH gradient strips (pH range, 3 to 11; Amersham) before isoelectric focusing on MULTIPHOR II apparatus (Amersham). After equilibration of the strip in equilibration buffer the second dimension was performed by standard gel electrophoresis using a Bis-Tris 8-12% gel. The gels were subsequently stained using colloidal blue (Invitrogen). The squares mark the approximate areas enlarged in B. **(B) **Enlargement of regions of the Ax2^bsR ^and *cdk8*^-^_*2 *_gels showing levels of the p42 and p32 proteins. The three paired biological repeats used in later analysis are shown. **(C) **The Imagemaster 2-DGE Platinum software (Amersham) was used to quantify the intensity of the p42 and p32 protein features as a 3-dimensional graphic. The height of each peak is representative of the intensity of each protein feature. The three biological replicates of each strain are shown.

In order to identify targets regulated by the Cdk8 protein, whole-cell extracts from vegetatively growing *cdk8*^-^_2 _and Ax2^bsR ^strains were analysed by two dimensional gel electrophoresis and gels were stained with colloidal blue. Subsequent analysis of the protein spots found that the vast majority of protein features showed equal staining intensities in both strains (Figure 5A). This characteristic was used to normalise the staining intensity of each gel against a standard. More detailed analysis found that two protein features were reproducibly altered in the *cdk8*^-^_2 _strain in comparison with the Ax2^bsR ^strain (Figure 5B and 3C). A protein approximately 42kDa (p42) in size was present at higher levels in the *cdk8*^-^_2 _strain, whilst a smaller protein of 32kDa (p32) was consistently expressed at lower levels in the mutant strain (Figure 5C).

These proteins were both possible targets of Cdk8-dependent regulation so were each excised from the gel and sequenced by mass spectrometry. This approach identified p42 as 4-hydroxyphenylpyruvate dioxygenase (HPD, dictybaseID number: DDB_G0277511). This metabolic enzyme is involved in catalyzing the conversion of 4-hydroxyphenylpyruvate to homogentisate in the tyrosine catabolic pathway [18-20]. The p32 protein was identified as the protein product of gene DDB_G0290659. BLAST searches against the NCBI database revealed that this protein shares a large amount of similarity with short-chain dehydrogenase/reductases from a number of different species. It was thus named SDR1 (short-chain dehydrogenase/reductase 1), while the gene encoding it was named *sdrA*.

### Expression of the *hpd *and *sdrA *genes

**Figure 6:**
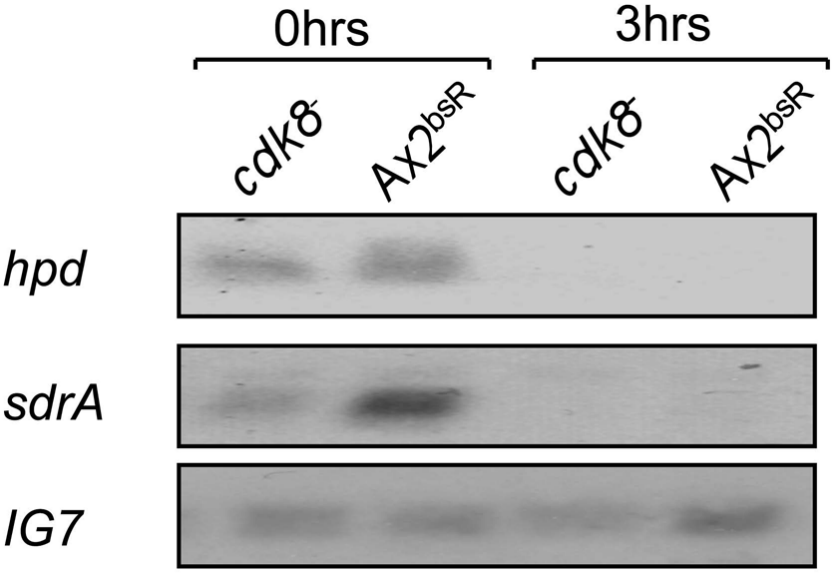
**Expression of *hpd *and *sdrA *in *cdk8*^-^_2 _cells**. Cells were harvested after vegetative growth (0hrs) or development on KK_2 _agar for 3hrs at 3 x 10^6 ^cells/cm^2^. RNA was extracted from these samples and resolved on a 1% formaldehyde gel, transferred to a nylon membrane and probed with ^32^P labelled fragments of the *hpd *and *sdrA *genes. The blot was reprobed with a ^32^P labelled fragment of the *IG7 *gene to control for loading.

As Cdk8 is a known transcriptional regulator, it was investigated whether the altered abundance of the HPD and SDR1 proteins were mirrored by alterations in the expression of the *hpd *and *sdrA *genes. RNA was extracted from Ax2^bsR ^and *cdk8*^-^_2 _cells that were growing vegetatively or had been developed on KK_2 _agar for 3hrs. Northern blot analysis of these samples indicated that *hpd *mRNA was present at equal levels in vegetatively growing *cdk8*^-^_2 _and Ax2^bsR ^strains and could not be detected in either strain after three hours of development (Figure 6). Examination of *sdrA *mRNA levels revealed that this transcript was present at higher levels in the Ax2^bsR ^cells than in *cdk8*^-^_2 _cells during vegetative growth (Figure 6). As with *hpd *mRNA no transcript could be detected after 3hrs of development in either strain. These observations implied that the reduced abundance of the SDR1 protein in the *cdk8*^-^_2 _strain may be the result of reduced expression or stability of the *sdrA *transcript, while the altered level of HPD was not due to a direct effect on mRNA levels.

## Discussion

The original *cdk8*^-^_HL _strain exhibited a complex phenotype which included defects in growth and aggregation. When developed under permissive conditions, the block upon aggregation was relieved but the resultant fruiting bodies were highly defective with no mature spores [11]. All of these defects could be complemented by expressing wild type Cdk8, but not a kinase dead version, from its endogenous promoter, so were dependent on Cdk8 activity. As a consequence of these observations, it was proposed that Cdk8 may function as a regulator of cell differentiation. A potential role for Cdk8 later in development was also suggested by a reduction in the proportion of Cdk8 associated with a high molecular weight complex late in development in response to an increase in extracellular cAMP [21].

Microarray analysis of genomic DNA revealed duplications in the genome of the *cdk8*^-^_HL _strain. The analysis strongly suggests that the *cdk8*^-^_HL _strain contains a large duplication of approximately 1.2 Mb on chromosome 2 and another smaller duplication of approximately 300 kb on chromosome 5. Chromosome 2 is the largest of the *Dictyostelium *chromosomes and a number of previous reports have suggested that it may be intrinsically unstable: In the NC4 strain originally isolated by John Bonner and maintained in vegetative growth for many decades, the chromosome is maintained as two smaller fragments [22]. Similarly the Ax4, but not the Ax2 strain, possesses a perfect inverted repeat of 1.5 Mb on chromosome 2 that has been proposed to be the result of a 'breakage-fusion-bridge' cycle [23]. The putative chromosome 2 duplication detected in the *cdk8*^-^_HL _strain is a similar size and occurs in a similar region of the chromosome as the Ax4 duplication. A number of other reports of chromosomal abberations in this region of the genome imply that it may be a hotspot for rearrangements [14]. The frequent occurrence of duplications in many strain backgrounds suggests that the segmental duplication on chromosome 5 in the *cdk8*^-^_HL _strain was not caused by genome instability resulting from loss of Cdk8 function.

Due to the *cdk8*^-^_HL _chromosomal abnormalities it was decided to create a new *cdk8 *disruptant. Characterisation of the newly generated mutant strain indicated that it had similar growth and aggregation defects as had previously been observed in the *cdk8*^-^_HL _strain [11,12]. Expression of myc-Cdk8 but not myc-Cdk8^kd ^rescued the growth and early development defects of the *cdk8*^-^_2 _strain indicating that these phenotypes were a direct consequence of an absence of Cdk8 kinase activity. PKA is a central regulator of *Dictyostelium *development and its activity has been found to be essential to the expression of a number of pre-aggregative genes including *aca *and *carA *[24]. As expression of *pkaC*, *aca *and *carA *could not be detected in *cdk8*^- ^cells [11] it was suggested that Cdk8 may exert its function upstream of PKA, so a FLAG-tagged PKA-C protein was over-expressed in both the *cdk8*^-^_*2 *_and Ax2^bsR ^strain. This approach results in constitutively active PKA and has been used to rescue aggregation defects in a number of strains including those deficient in ACA, Erk2 and AmiB [25-27]. FLAG-PKA-C protein expression in the Ax2^bsR ^strain resulted in rapid development; a phenotype reported to be a consequence of high PKA activity [17], consistent with the production of active protein. However, although expressing similar levels of FLAG-PKA-C as detected by western blot, the *cdk8*^-^_2_[*act15*/FLAG-*pkaC*] strain exhibited similar defects as the *cdk8*^-^_2 _strain implying that defects in aggregation were not merely a consequence of defects in PKA regulated gene expression. It is possible that the levels of PKA-C expression were not high enough to rescue the defect, despite its expression from a multicopy extrachromosomal vector using a strong promoter.

The *cdk8*^-^_2 _aggregation deficiency is comparable to that observed in a strain deficient in AmiB [27]. These phenotypic similarities might be expected as AmiB has been identified as a *Dictyostelium *homologue of Med13, a component of the same Mediator module as Cdk8 [8]. However, the observation that constitutive PKA activity rescues the *amiB*^- ^[27] but not the *cdk8*^-^_2 _aggregation defect is interesting as it implies that AmiB and Cdk8 operate in different signalling pathways. Such an observation would not be unprecedented as the Med13 and Cdk8 proteins have been shown to play subtly different roles during *Drosophila *development [8]. However, we cannot rule out that the precise level of overexpression of PKA-C is important in rescuing the phenotype.

Although the phenotypes of the *cdk8*^-^_2 _and *cdk8*^-^_HL _cells appeared identical with regard to growth and early development, this was not the case during later development. It was previously observed that the *cdk8*^-^_HL _strain formed aberrant fruiting bodies which contained virtually no viable spores. This late developmental defect was accompanied by a decrease in expression levels of the spore-specific transcript *spiA *[11]. In contrast, the newly generated *cdk8*^-^_2 _strain produced phenotypically normal fruiting bodies in which the *spiA *transcript was expressed at normal levels. Microscopic examination of disaggregated *cdk8*^- ^fruiting bodies revealed that the strain was capable of producing morphologically normal spores (data not shown). However, these spores exhibited only 40% viability when compared to those formed by the Ax2^bsR ^control strain. Endogenous expression of a myc-Cdk8 protein rescued this defect in spore viability indicating that it was a consequence of loss of Cdk8 function. These data suggested that Cdk8 is involved in *Dictyostelium *spore cell differentiation in cells that are presumed to lack the genomic duplications present in the *cdk8*^-^_HL _strain. However, the role is more subtle than when these duplications, and perhaps other unknown smaller-scale mutations, were present.

A 2D-PAGE comparison of the *cdk8*^-^_2 _and Ax2^bsR ^strains was unable to detect any differences in the abundance of the vast majority of proteins. This is consistent with microarray analysis in which it was observed that Srb10 was involved in the regulation of only 3% of *S. cerevisiae *genes [2]. Despite the similarities between the *cdk8*^-^_2 _and Ax2^bsR ^proteomes, two proteins were consistently observed to be present at different levels in the *cdk8*^-^_*2 *_strain. One of these proteins was abnormally abundant in *cdk8*^-^_*2 *_cells and was identified as the *Dictyostelium *homologue of 4-hydroxyphenylpyruvate dioxygenase (HPD). The other protein, named SDR1 exhibited homology to the short chain reductase proteins and was found to be present at unusually low levels in the *cdk8*^-^_2 _strain.

Short chain dehydrogenases are a large family of NAD or NADP-dependent oxidoreductases working on a variety of substrates with short carbon chains. For example alcohol dehydrogenases break down alcohols which could be toxic and are also involved in generating aldehydes and ketones during various biosynthetic processes. In bacteria and yeast this enzyme can be used to synthesize alcohol in anaerobic conditions. It is not possible to predict the substrate of the *Dictyostelium *SDR1 from sequence homology. The SDR1 protein has been found to become associated with phagosomes during maturation [28] and expression of the *sdrA *gene was also found to be upregulated as part of the *Dictyostelium *response to Legionalla infection [29]. HPD is a metabolic enzyme involved in catalyzing the conversion of 4-hydroxyphenylpyruvate to 2,5 dihydroxyphenylacetic acid (homogentisate or melanic acid) as part of the pathway to degrade tyrosine and phenylalanine [18-20]. In *Dictyostelium, *the HPD protein has been found to associate with the centrosome [30] and has been implicated in phagosomal maturation [28]. Microarray analysis revealed that the *hpd *gene is upregulated in the environmentally resistant aspidocyte cell type that is formed by *Dictyostelium *in response to stress [31]. Both of these proteins are catabolic enzymes found associated with phagosomes and are implicated in stress responses. This would fit for a general role for Cdk8 in regulating stress responses, consistent with data from *S. cerevisiae *showing regulation of cyclin C in response to environmental stresses [3]. Microarray analysis of *S. cerevisiae *deficient in *srb10 *(the cdk8 orthologue) showed altered expression of around 3% of yeast genes and half of these are genes derepressed during nutrient starvation [2], again consistent with a general role for Cdk8 in mediating metabolic responses.

Despite the difference in protein levels, the *hpd *mRNA was present at similar levels in the two strains. However the *sdrA *transcript was found to be significantly less abundant in *cdk8*^-^_2 _cells than in the Ax2^bsR ^strain. This suggested that the lower levels of the SDR1 protein in the mutant cell line may be due to defects at the transcriptional level whereas the effect on HPD levels is post-transcriptional. The established role for Cdk8 in transcriptional regulation via the mediator complex would suggest that the *sdrA *gene is a candidate for direct transcriptional regulation by Cdk8 in *Dictyostelium*.

## Conclusions

This analysis has confirmed the importance of Cdk8 at multiple stages of *Dictyostelium *development, although the severity of the defect in spore production depends on the genetic background. Potential targets of Cdk8-mediated gene regulation have been identified in *Dictyostelium *which will allow the mechanism of Cdk8 action and its role in development to be determined.

## Methods

### Growth, development and strain generation of *Dictyostelium*

*Dictyostelium *cells were grown axenically in HL5 medium at 22°C in shaking suspension. For development in shaking suspension, exponentially growing cells were resuspended in KK2 (16.5 mM KH_2_PO_4_, 3.8 mM K_2_HPO_4_) at 2 x 10^7 ^cells/ml and shaken at 120 rpm and 22°C for 5 hours, pulsed with 50 nm cAMP every 5 minutes. For filter development exponential axenically growing cells were washed in KK_2 _and re-suspended in LPS (40 mM KH_2_PO_4_, 20 mM KCl, 680 μM dihydrostreptomcin sulphate [pH7.2]), pulsed as above and plated at 3.5 x 10^6^/cm^2 ^on Millipore filters on an LPS soaked pad. The filters were incubated at 22°C in the dark.

The entire *pkaC *coding sequence was amplified by PCR using primers to introduce a FLAG tag sequence at its 5' terminus. This fragment was inserted into the BamH1 and Xho1 sites of pDXA-3C vector [32] to generate pDXA[*act15*/FLAG-*pkaC*] plasmid. The cdk8KO-pLPBLP vector was constructed to allow disruption of the *cdk8 *locus in Ax2K by the insertion of a blasticidin resistance (bsR) cassette. This vector contained the same regions of *cdk8 *homology as the pCDK8-KOI vector that had been used to create the original *cdk8*^- ^strain [11] but used the pLPBLP vector [33] as its backbone. Constructs were introduced into *Dictyostelium *Ax2 cells by electroporation and transformants selected by growth in the presence of G418 (10 μg/ml) or Blasticidin (5 μg/ml) as appropriate. All strains were generated with the approval of the Biochemistry Department Genetic Modification Safety Committee, University of Oxford. The strain used for the phenotypic analysis in this manuscript will be lodged as a communal resource in the *Dictyostelium *Stock Center (dictybase.org).

### Northern analysis

Total RNA was extracted from approximately 1 x 10^7 ^cells using TRIZOL RNA extraction kit (Sigma) according to the manufacturer's protocol. Samples (10 μg) of total RNA were separated on a 1% formaldehyde-containing gel, blotted and probed by standard methods.

### Analysis of genomic DNA by microarray

A protocol similar to that used to analyse RNA was used to compare genomic DNA (gDNA) by microarray. 5 μg of genomic DNA in T0.1E buffer (10 mM Tris-HCl, 0.1 mM EDTA, pH 8) was sonicated. 20 μl of 2.5X random primer mix (Bioprime kit, Invitrogen) was added and the mixture heated for 5mins at 95°C before being placed on ice. 5 μl of 10x dNTP mix (1.2 mM dATP, dTTP, dGTP and 0.6 mM dCTP in T0.1E), 3 μl of Cy5-dCTP or Cy3-dCTP (final concentration 60 μM) and 1 μl of Klenow fragment (40U/μl, Invitrogen) was then added and the reaction mixture incubated, away from light at 37°C. After 2hrs the reaction was stopped by the addition of 5 μl of 0.5 M EDTA (pH 8). The labelled gDNA was separated from other reaction components by passing the reaction mixture through a G-50 column. This gDNA was then precipitated, hybridised to the microarray slide overnight at 42°C [34]. Arrays were scanned using an Axon Instruments GenePix 4000B scanner and fluorescence quantified using the GenePix 3.0 software. Subsequent data processing steps were carried out using the limma package, part of the Bioconductor project, using the R statistical environment [35-37]. Background fluorescence was subtracted using the method of Kooperberg et al [38] and data were then normalised using the print-tip loess algorithm. Single hybridisations were sufficient to identify large segmental duplications. The array data has been deposited in ArrayExpress under the accession E-TABM-1013. The array design, also available from ArrayExpress, has the accession A-SGRP-3.

### Two dimensional (2D) gel electrophoresis

Aliquots of whole cell extracts containing 100 μg of soluble protein were acetone precipitated and the pellets resuspended in 125 μl of sample buffer (5 M urea, 2 M thiourea, 4% 3-[(3-cholamidopropyl0-dimethylamminio]-1-propanesulfonate (CHAPS), 4% dimethylbenzylammonium propane sulfonate (NSDB-256), 1% tributylphosphine (TBP), 1% dithiothreitol, 10 mM benzamidine, 1 mM sodium orthovanadate, 1 mM sodium fluoride and trace amounts of bromophenol blue). The samples were absorbed into a nonlinear immobilized pH gradient strip (pH 3-11, Amersham) and isoelectric focusing performed using MULTIPHOR II apparatus (Amersham) according to the manufacturer's guidelines [39]. Each strip was then immersed in equilibration buffer (4 M urea, 2 M thiourea, 2% DTT, 2% SDS, 0.05 M Tris [pH 6.8], 30% glycerol, and trace levels of bromophenol blue). Proteins absorbed into the strip were then resolved according to size using standard electrophoresis on NuPage bis-Tris 4-12% ZOOM gels (Invitrogen). Gels were subsequently stained with colloidal blue (Invitrogen) and features analyzed using ImageMaster 2-DGE Platinum software (Amersham).

### Protein identification

Protein spots were cut from the gel, digested with Trypsin and peptide masses were identified by Tandem Mass Spectroscopy (MS-MS) [39]. Peptide masses were compared to protein databases (DictyBase, NCBI, SwissProt) using BLAST for protein identification.

## Authors' contributions

DG carried out the genetic and proteomic work and helped draft the manuscript. GB, JS and AI designed and prepared microarrays, GB and JS carried out and analysed microarray experiments and prepared the relevant figures. CP conceived the study, participated in its design and helped draft the manuscript. All authors have read and approved the final manuscript.
